# Society Meetings

**Published:** 1910-12

**Authors:** 


### SOCIETY MEETINGS

---

New York and New England Association Railway Surgeons held its twentieth annual session at the Hotel Astor, New York, November 3 and 4, 1910, under the presidency of Dr. L. M. Bingham, of Burlington, Vt., chief surgeon of the Rutland Railroad. Dr. John B. Deaver, of Philadelphia, delivered the address in surgery.

The following officers were elected for the ensuing year: President, F. A. Goodwin, Binghamton, N. Y.; first vice-president, Walter Lathrop, Hazelton, Pa.; second vice-president, John W. Le Seur, Batavia, N. Y.; corresponding secretary, George Chaffee, Brooklyn, N. Y.; recording secretary, C. A. Pease, Burlington, Vt.; treasurer, J. K. Stockwell, Oswego, N. Y.

---

The American Academy of Medicine (specialising in medical sociology) will hold its fourth mid-year meeting, being a conference on the sociologic influence of hospitals and dispensaries, at the Lafayette Hotel, Buffalo, Thursday and Friday, December 1 and 2, 1910. Under the general topic, "The Economic Influence of Hospitals," the following papers will be read: "Does the Public Hospital Afford Sufficient Recompense to the Staff for its Services?" Dr. Thomas D. Davis, Pittsburgh; "The Economic Influence of Hospital Administration on the Medical Profession," Dr. A. Vander Veer, Albany. Other papers on this and kindred topics will be read.

---

The Rochester Academy of Medicine held meetings during the month of November as follows:

*Section of General Medicine.*—Wednesday evening, November 2. Program: The use of the *x*-ray in medical diagnosis, H. M. Imboden, Clifton Springs, N. Y.

*Section of Surgery.*—Wednesday evening, November 9. Program: Drainage of the gall-bladder high up under the costal arch, F. W. Zimmer; Operations during pregnancy, John F. W. Whitbeck; Report of operative cases of gall-bladder infection following typhoid fever, E. W. Mulligan.

*Section of Obstetrics, Gynecology, and Pediatrics.*—Wednesday evening, November 16. Program: Report of cases treated with vaccine, Wesley T. Mulligan.

*Section of Public Health.*—Wednesday evening, November 23. Program: Some problems in sewage disposal, Professor Charles Wright Dodge.

---

The Buffalo Academy of Medicine held meetings during the month of November, as follows:

*Section of Surgery.*—Tuesday evening, November 1. Program: Dilatation of the urethra, Charles W. Bethune; "Man's inhumanity to man," lantern slide illustrations of medieval torture methods, Roswell Park.

*Section of Medicine.*—Tuesday evening, November 15. Program: Report of committee on epidemic poliomyelitis, Irving M. Snow, chairman; Epidemic poliomyelitis, Bernard Sachs, M.D., Chairman of the New York Committee on Epidemic Poliomyelitis, Neurologist to the Bellevue and Mt. Sinai Hospital, New York. Discussion by J. W. Putnam, Irving M. Snow, F. S. Crego and others.

*Section of Pathology.*—Tuesday evening, November 22. Program: Symposium on pathology of the thyroid; observations on goitre in fish and the relation of goitre to water, Harry Gaylord; Clinical pathology and therapy of thyroid, P. S. Beebe, Professor and Examiner of Therapeutics, Cornell University.

---

The Southern Surgical and Gynecological Association, will hold its twenty-third annual meeting at Nashville, December 13, 14, and 15, 1910, under the presidency of Dr. William O. Roberts, of Louisville. The new Hotel Hermitage has been selected as headquarters and the meetings will be held in the hotel assembly room. Dr. Rufus E. Fort is chairman of the committee of arrangements; and Dr. William D. Haggard, 148 Eighth Avenue, Nashville, is the permanent secretary. The preliminary program contains a list of fifty papers, the authors of twenty being from the north.

---

The Medical Association of Central New York held its forty-third annual meeting at Syracuse, October 19 and 20, 1910, at which time the following named were elected officers: president, Wesley T. Mulligan, Rochester; vice-president, William G. Johnson; secretary, John J. Beuttner, Syracuse; treasurer, Charles O. Boswell, Rochester. The next meeting will be held at Rochester.

---

THE Eighth District Branch of the Medical Society of the State of New York held its annual meeting at Buffalo, September 30, 1910, at which time the following named were elected officers: president, Thomas H. McKee, Buffalo; vice-presidents, Henry A Eastman, Jamestown, and Arthur G. Bennett, Buffalo; secretary, Carl S. Tompkins, Buffalo; treasurer, Charles A. Wall, Buffalo.

